# Resonant laser excitation for nanoscale photocatalytic gold growth on patterned templates

**DOI:** 10.1038/s41598-026-36556-5

**Published:** 2026-01-19

**Authors:** Jan Schardt, Moritz Paulsen, Fatemeh Abshari, Martina Gerken

**Affiliations:** Integrated Systems and Photonics, Faculty of Engineering, Kiel University, Kiel, Germany

**Keywords:** Photocatalysis, UV laser excitation, Photoluminescence, Nanograting structures, Resonant waveguides, Neuromorphic computing, Chemistry, Materials science, Optics and photonics

## Abstract

Resonant excitation of nanooptical templates offers great advantages for localized photocatalytic activity. We present a design, fabrication and characterization of nanostructured TiO_2_ to mimic the dynamic behavior of growth of long-range axonal connections in neuromorphic computing architectures. We use the photocatalytic activity of the TiO_2_ to reduce gold particles from a precursor solution and influence the localized gold growth with light-dependent field enhancements inside the TiO_2_. Nanooptical templates with different grating parameters are suggested to individually control the formation of metal lines on the surface. We show with an optical probe that resonant excitation with a UV laser is achieved with the nanostructures. 150 nm of DPVBi is used as an emissive layer on top of the patterned TiO_2_ to visualize the field enhancement. The experiments show an emission increase for selected grating periods depending on the excitation setup. For nanostructures with only a few lateral repetitions, the quality factor of the resonance wavelength is lower which leads to a broader range of grating periods that resonantly increase the emission of DPVBi. We perform growth experiments and find dense coverage of gold particles on the nanostructured lines, if excited resonantly. For non-resonant excitation and on planar TiO_2_ areas, gold particles are sparsely scattered. These results show that local manipulation of dynamic growth characteristics is achieved.

**Supplementary Information:**

The online version contains supplementary material available at 10.1038/s41598-026-36556-5.

## Introduction

The ability to precisely control the layer formation of metal lines locally is a critical aspect of advancing nanotechnology, particularly in applications such as sensing^1,2^, neuromorphic computing^3,4^, and optoelectronics. Often times, standard lift-off lithography techniques^5–7^ or etching processes^8^ are used to create controlled structures. Photocatalysis is a highly dynamic mechanism that already offers great advantages and is used in a variety of applications^9,10^. Growth of metals from a precursor solution by photocatalysis offers a dynamic process to design conductive shapes on a given surface that can be dissolved chemically to reverse the formation^11^.

Photocatalytically grown metal lines may offer a promising approach for mimicking certain aspects of axonal growth and pruning in neuromorphic computing architectures^12,13^. Gold nanoparticles, in particular, have attracted significant attention due to their unique optical and photocatalytic properties, making them relevant for use in such systems^14–16^. We investigate the growth process on the surface of photoactive titanium dioxide (TiO_2_) for its excellent photocatalytic activity^17,18^. Using a dielectric interface to promote gold growth on its surface was shown in previous publications^19,20^. Further, it was shown that illuminating through a shadow mask allows for localized gold growth on TiO_2_ layers^21^ as well as micro-structuring the TiO_2_ to confine the growth to a specific area^22,23^. While this study does not directly demonstrate neuromorphic functionality, the explored growth mechanisms are inspired by and potentially applicable to those architectures.

In this research, we investigate the use of resonant laser excitation to enhance the photocatalytic growth probability locally on patterned nanoscale templates. Laser assisted photocatalysis was studied before without the use of nanograting structures^24^. Our approach allows for a precise spatial control of the growth provided by the design of the template in combination with the excitation light and angle. Nanograting structures on high refractive index layers allow to selectively guide light into the nanostructure that lead to locally increased field intensities^25,26^. It was shown that nanograting structures excited with a monochromatic laser offer a unique tuneability of the desired resonance wavelength depending on the excitation wavelength and its angle of incidence as well as the parameters of the grating structure^27^. With this approach, we aim for a technical representation of a stimulus-dependent formation of neuronal connections with the growth of metal lines under specific conditions. We vary the grating parameters and investigate the field enhancement regarding its sensitivity to different excitation conditions. To visualize the local field enhancement, optical probes are employed^28–30^. This paper presents the design choices of the nanooptical template, the characterization setup and experiments with resonant laser-induced gold formation on nanopatterned TiO_2_.

### Design of the nanostructure template

**Fig. 1:**
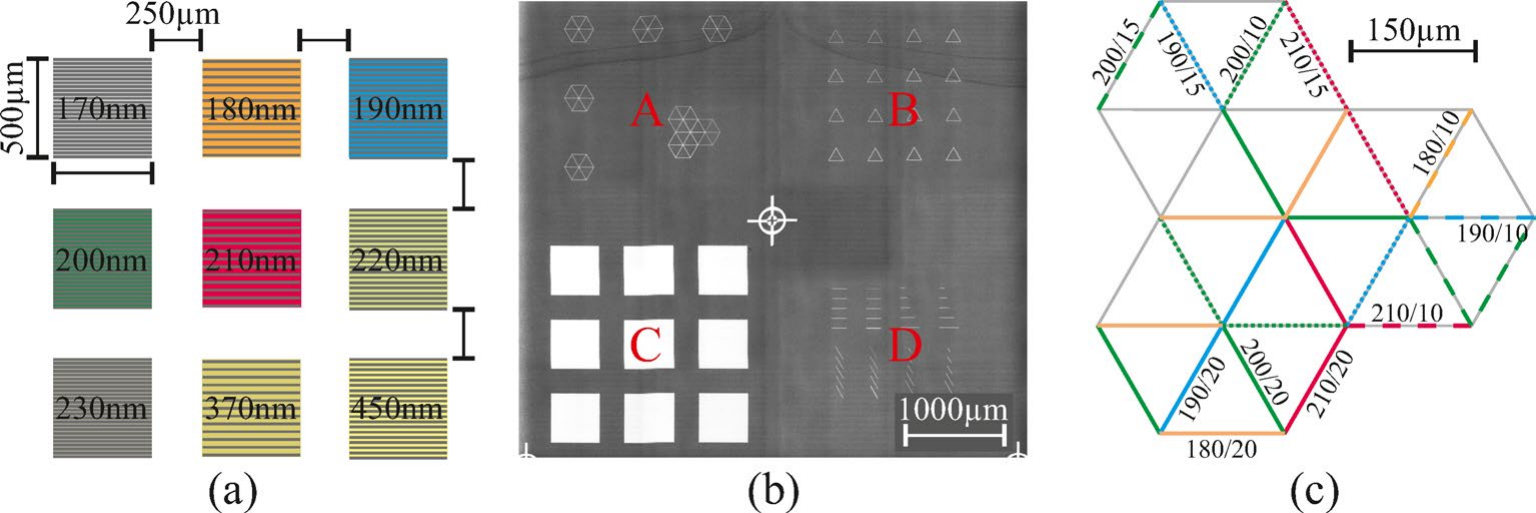
**(a)** Schematic of the square nanostructures in field C with grating periods ranging from $\:{\Lambda\:}=170-230\:\mathrm{n}\mathrm{m}$ with two additional grating periods for the visible regime as reference. **(b)** Laser interference microscope image of the master template; field A-D are denoted red. **(c)** Schematic of the hexagonal structure in field A; the grating period and repetition number is denoted on the nanostructure lines as $\:{\Lambda\:}/r$ in nm. The color coding matches **(a)**.

The nanooptical template consists of different types of nanostructure patterns and we refer to them as field A, B, C and D. A laser interference microscope image (Keyence VK-X260-K) of the fields and designs is depicted in Fig. 1 (b). Field A and B resembles the network of an artificial neuronal network with multiple nodes and interconnecting lines with 150 μm length, where field A has hexagonal structures and field B triangular structures, respectively. Field C consists of large nanostructured squares and field D has multiple nanostructure lines at different orientations and lengths.

The design for the grating structure considers the photocatalytic gold-growth requirements. The experiments are carried out with UV illumination and we choose the grating dimensions to match the excitation wavelengths of $\:\lambda\:\approx\:350\:\mathrm{n}\mathrm{m}$. So-called quasi-guided modes (QGM) are coupled by the grating structure and the resonance wavelengths $\:m\cdot\:{\lambda\:}_{m}$ are calculated in dependency of the grating period $\:{\Lambda\:}$, the effective refractive index of the stack $\:{n}_{\mathrm{e}\mathrm{f}\mathrm{f}}$, the refractive index of the surrounding medium $\:{n}_{\mathrm{i}\mathrm{n}\mathrm{c}}$ and the angle of incidence $\:{\vartheta\:}_{\mathrm{i}\mathrm{n}\mathrm{c}}$ with $\:m\in\:\mathbb{N}$:

1 $$\:m\cdot\:{\lambda\:}_{m}=\:{\Lambda\:}({n}_{\mathrm{e}\mathrm{f}\mathrm{f}}\left(\lambda\:\right)\mp\:{n}_{\mathrm{inc}}\mathrm{sin}\left({\vartheta\:}_{\mathrm{i}\mathrm{n}\mathrm{c}}\right))$$

We aim to cover a variety of different grating periods $\:{\Lambda\:}$, grating repetitions $\:r$ and orientation angles on the template to study their influence on resonant coupling of incident light thus a changing behavior in the photocatalytic activity. We preliminarily estimated effective refractive indices between 1.6 and 2.1 refractive index units (RIU) depending on the layer thickness of the TiO_2_ layer which lead to a variation of grating periods ranging from $\:{\Lambda\:}=170\:\mathrm{n}\mathrm{m}$ to $\:{\Lambda\:}=230\:\mathrm{n}\mathrm{m}$. A nominal grating depth of 45 nm was targeted for high quality factor resonances^31^. These grating periods are used on all four fields and are varied in their orientation and repetition. In preliminary simulations we found that nanostructures with grating repetitions as low as $\:r=10$ are able to guide modes in the waveguide. Thus, we vary the number of repetitions for the hexagonal-, triangular- and line-shaped nanostructures from $\:r=10$ to $\:20$. The nanostructured squares’ dimensions are 500 μm by 500 μm. The grating repetition depends on the grating period of the squares. Additionally, two squares with grating periods $\:{\Lambda\:}=380\:\mathrm{n}\mathrm{m}$ and $\:450\:\mathrm{n}\mathrm{m}$ for visible resonance wavelengths are added as visual reference and quality control during fabrication.

## Experimental section

### Fabrication of nanooptical templates

All samples in this work are prepared on 25 × 25 mm² soda-lime glass substrates with a thickness of 1.1 mm. The nanograting structures are fabricated based on a standard UV nanoimprint lithography process^32^. The master templates according to our design were fabricated via electron-beam lithography (Kelvin Nanotechnology Ltd.). On the 25 × 25 mm² area all four previously mentioned nanograting designs A-D shown in Fig. 1 are available four times. This layout allows to characterize and use all designs with the same fabrication conditions multiple times and compare the designs with each other.

**Fig. 2:**
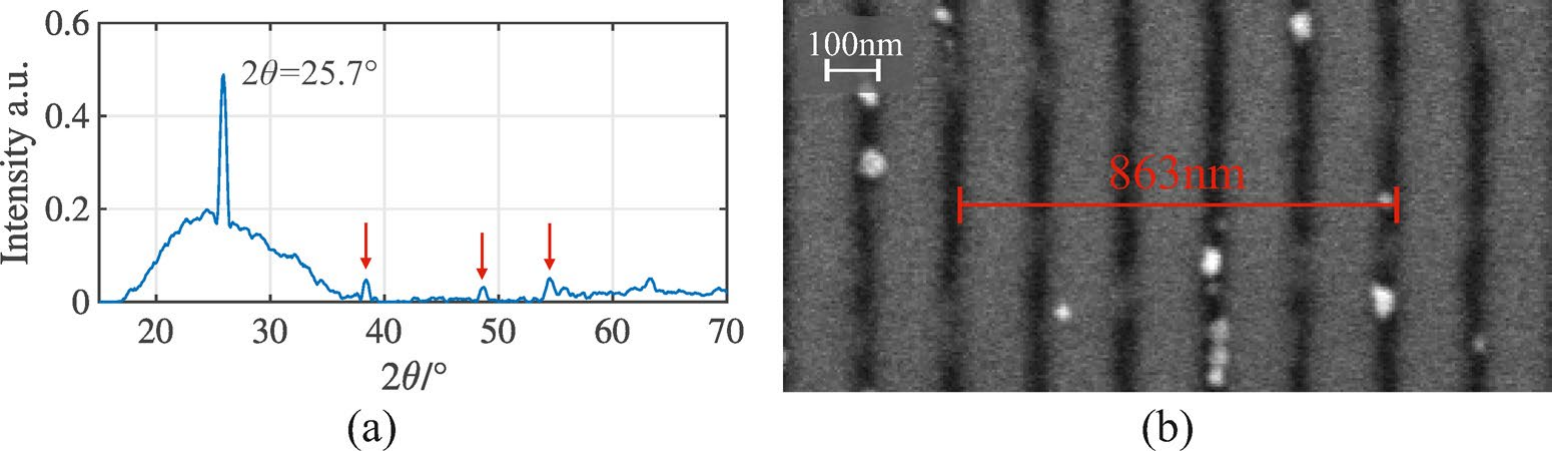
**(a)** XRD characterization of the annealed TiO_2_ with major reflections at $\:2\theta\:=25.7^\circ\:,\:38.2^\circ\:,\:48.7^\circ\:$ and $\:54.5^\circ\:$. The smaller less visible peaks are indicated with the red arrows and the intensity is normalized to the maximum. **(b)** SEM image of the $\:{\Lambda\:}=170\:\mathrm{n}\mathrm{m}$ grating; the measured distance across five grating periods is 863 nm.

First, a negative imprint template is created using polydimethylsiloxane (PDMS; Dow, Sylgard 184 with a curing agent in an 8:1 ratio) from the master template structure. The UV imprint resist Amonil MMS4 (AMO GmbH, Aachen, Germany) is then spin-coated onto a cleaned glass substrate at 3000 rounds per minute. The resist layer thickness was measured to be $\:\approx\:200\:\mathrm{n}\mathrm{m}$ and the PDMS stamp is manually pressed into the resist. After UV curing, the PDMS is removed, transferring the nanostructure onto the glass substrate. Next, a thin layer of 100 nm TiO_2_ (Kurt J. Lesker, EJUTIO2403TK4) is deposited on the nanostructures via sputter deposition. TiO_2_ is selected for its outstanding photocatalytic properties, particularly in the anatase phase^33–35^. To promote the anatase phase, the sample is annealed at 400 °C for 90 min^36^. As confirmed by XRD in Fig. 2 (a), the annealed TiO₂ exhibits the characteristic anatase reflections at $\:2\theta\:=25.7^\circ\:,\:38.2^\circ\:,\:48.7^\circ\:$ and $\:54.5^\circ\:$, with the corresponding raw data provided in the Supplementary Information^37^. The samples will be used for the experiments discussed in Sect.“Photocatalytic gold growth”.

To visualize the resonance effects due to the grating structures, we use 4,4 -bis(2,2 -diphenylvinyl)−1,1 -diphenyl (DPVBi) as an optical probe material. DPVBi is an organic emissive material in the blue visible region that is commonly used in the fabrication of organic light emitting diodes^38^. It is excited in the UV regime with wavelengths that are typically used for photocatalytic growth^39^. Its absorption maximum $\:{\lambda\:}_{\mathrm{a}\mathrm{b}\mathrm{s}}=351\:\mathrm{n}\mathrm{m}$ perfectly matches the excitation wavelength of the used laser. The nanostructured samples are transferred into a high vacuum evaporation system and a layer of 150 nm DPVBi (BOC-sciences, 142289-08-5) is coated on the TiO_2_-layer. After deposition the samples are characterized immediately since the organic material degrades in air and loses its photoluminescent (PL) characteristic over time. The evaporation and sputter parameters were calibrated in separate experiments using profilometry on reference samples. Under the conditions used here, the resulting TiO₂ film thickness is (100 ± 3) nm, and the DPVBi layer (150 ± 5) nm.

Scanning electron microscopy (SEM) reveals the nanoscale morphology of the $\:{\Lambda\:}=170\:\mathrm{n}\mathrm{m}$ grating after coating with the TiO₂ layer. The image is recorded with a Zeiss Ultra Plus microscope with an acceleration voltage of 5 kV and shown in Fig. 2 (b). The grating lines remain defined and continuous across the surface, demonstrating that the pattern is preserved after deposition. A line measurement across five periods yields a total distance of 863 nm, corresponding to an average period of 173 nm. Additional Field Emission SEM (FESEM) and atomic force microscopy (AFM) measurements of the nanostructures were carried out by Kelvin Nanotechnology Ltd. and are shown in the Supplementary Information (Supplementary Fig. 1,2). The SEM image shows a fill factor of $\:\approx\:45\%$. AFM measurements show the height profile extracted from a line scan across multiple periods and reveal a grating depth of 38 nm, slightly below the nominal targeted value of 45 nm. Both measurements confirm that the fabricated structures possess a well-defined periodic profile.

### Laser excitation setup

**Fig. 3:**
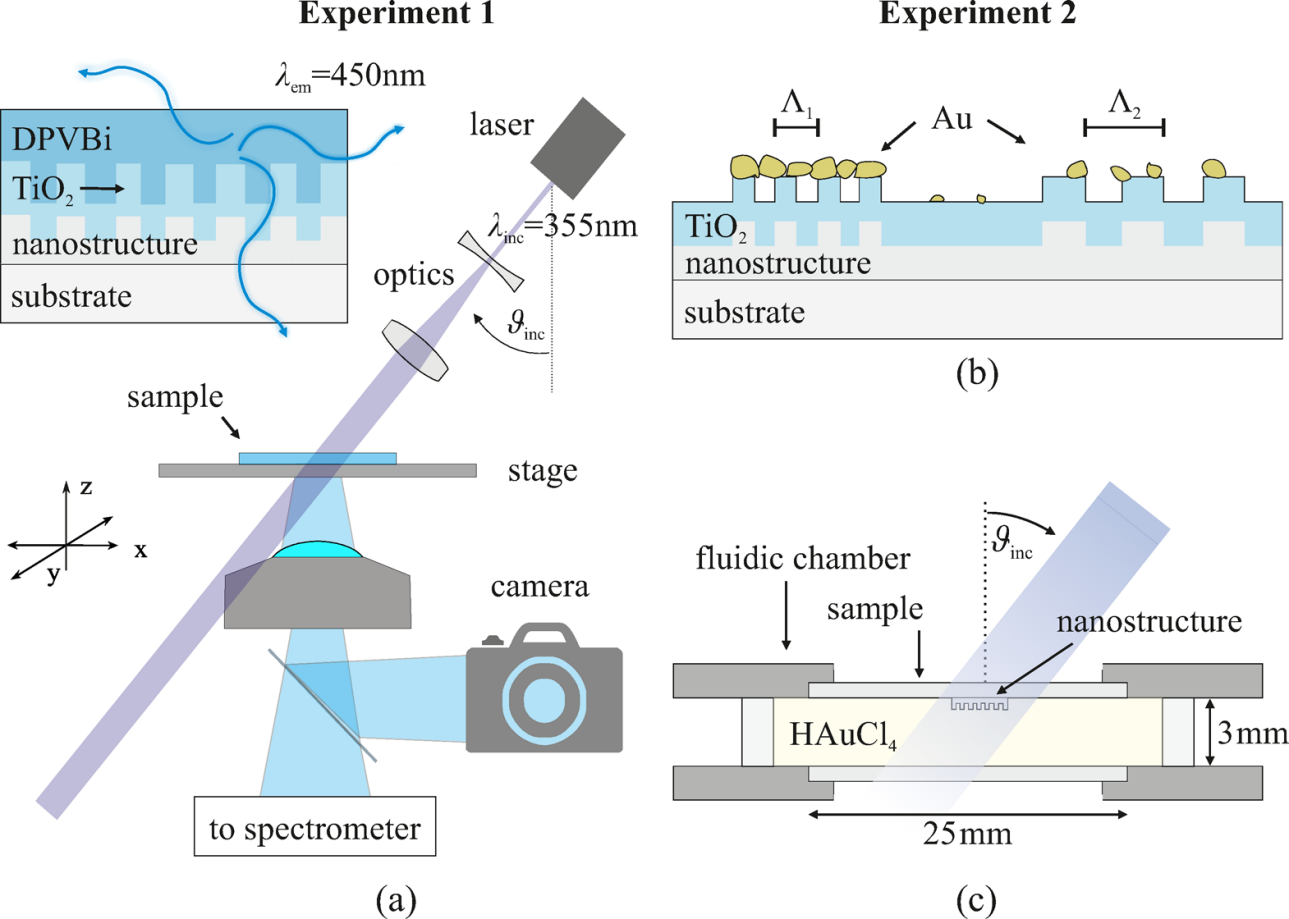
Schematics of both experiment setups. **(a)** Laser with $\:{\lambda\:}_{\mathrm{i}\mathrm{n}\mathrm{c}}=355\:\mathrm{n}\mathrm{m}$ and collimating optics transmits the sample placed on a xy-stage. Transmission images and spectra can be recorded with a camera and spectrometer, respectively. Experiment 1 shows the optical characterization of nanostructured samples with DPVBi emissive layer. **(b)** Schematic of a sample after the growth experiment with two different grating periods. **(c)** Nanostructured samples facing down inside a fluidic chamber filled with HAuCl_4_ solution; the nanostructure is excited locally with the laser. Images prepared with Corel Draw, Version 2024.

We decided to use a laser-based characterization setup for the samples and divide the following setups in two experiments as shown in Fig. 3. The first experiment setup is used to characterize the nanostructured samples with a DPVBi emissive layer, whereas experiment 2 utilizes the laser to promote gold growth in a fluidic chamber without an emissive layer. For both experiments, resonant coupling of incident light into waveguides via grating structures is achieved following the Bragg-Eq. (1). The setup depicted in Fig. 3 (a) uses a microscope (Nikon Ti-U) for image capture and a UV laser (Cobolt Zouk, 355 nm, 10 mW) for sample excitation. A UV laser is chosen to match typical excitation wavelengths for photocatalytic gold growth and its center wavelength $\:{\lambda\:}_{\mathrm{i}\mathrm{n}\mathrm{c}}=355\:\mathrm{n}\mathrm{m}$ matches the resonance wavelength expected with the given grating periods around $\:\:{\Lambda\:}=200\:\mathrm{n}\mathrm{m}$. The laser is positioned above the sample at a variable angle, and collimating optics are employed to expand the beam to a diameter of approximately 1.5 mm. The widened beam is essential to obtain homogeneous excitation throughout the nanostructured areas. The unpolarized laser beam transmits the sample without directly coupling in the objective lens of the microscope. The sample is placed on a motorized XY-stage and can be rotated in plane. This microscope setup combined with the stage and rotation allows for precise positioning and orientation of the sample with respect to the grating and incident direction of the laser and is used for both experiments.

To ensure that manually selected spots on the sample are properly excited, the laser must be centered within the camera’s field of view before each measurement or growth experiment. This is achieved by placing a fluorescent dummy sample in the setup, then adjusting the laser’s angle of incidence, and positioning such that the camera detects the fluorescence of the sample.

### Optical characterization

**Fig. 4:**
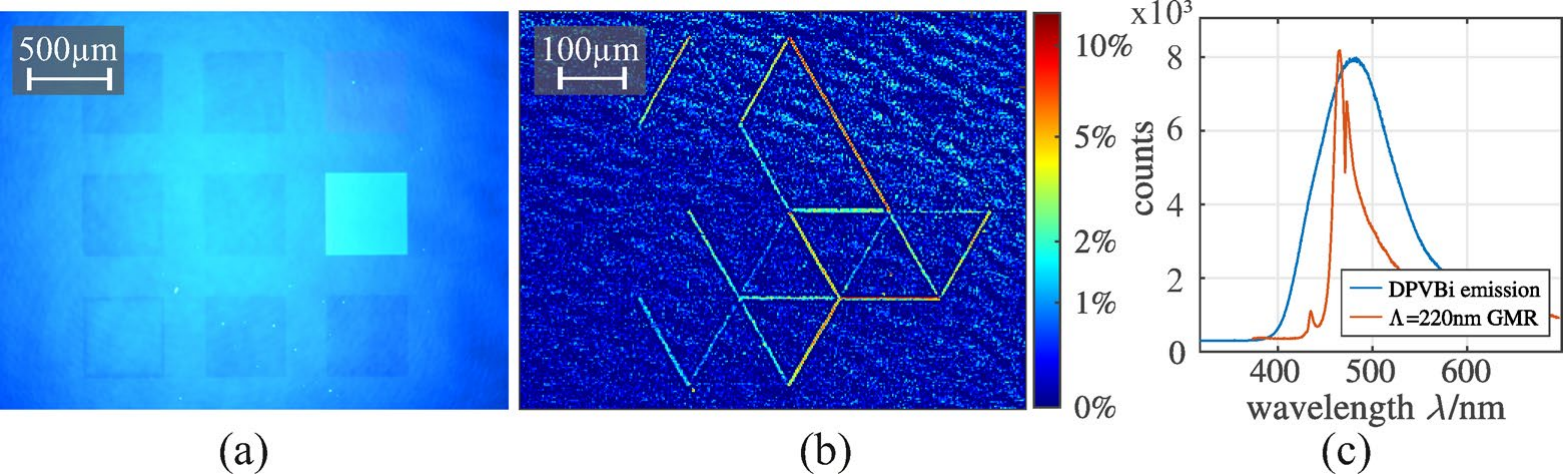
**(a)** Period-dependent PL enhancement measured on the square grating structures for 150 nm DPVBi on 100 nm TiO₂ under monochromatic excitation at $\:{\lambda\:}_{\mathrm{i}\mathrm{n}\mathrm{c}}=355\:\mathrm{n}\mathrm{m}$ and $\:{\vartheta\:}_{\mathrm{i}\mathrm{n}\mathrm{c}}=30^\circ\:$. The enhancement is most pronounced for $\:{\Lambda\:}=220\:\mathrm{n}\mathrm{m}$. **(b)** Background-normalized fluorescence map of the hexagonal grating structures revealing spatially resolved emission enhancements for $\:{\Lambda\:}>190\:\mathrm{n}\mathrm{m}$ with a maximum relative increase of $\:\approx\:10\%$ at $\:{\Lambda\:}=210\:\mathrm{n}\mathrm{m}$. The broader period range compared to the square geometry is consistent with the reduced number of grating repetitions and the associated resonance broadening. The corresponding raw fluorescence micrograph is provided in Supplementary Fig. 4. **(c)** Representative DPVBi emission spectrum on the nanostructured TiO₂ template together with the measured guided-mode resonance response.

The DPVBi-coated samples are characterized with the laser excitation setup described in Sect. “Laser excitation setup” with the lasers output power set to 2 mW. The emission spectrum of the DPVBi is recorded with a spectrometer. It is observed that the excitation wavelength of the laser is not visible in Fig. 4 (c), thus does not disturb the image acquisition with the camera. Further, pictures of the emission pattern of the nanostructure fields A and C are taken with the camera to observe the emission intensity locally. The different fields of the nanostructure design are excited separately.

### Photocatalytic gold growth

We investigate the enhancement of photocatalytic activity due to resonant coupling to the TiO_2_ waveguide and therefore conduct gold-growth experiments with the samples without the additional emissive layer, solely relying on the field enhancement due to resonant guiding. The sample is placed on the top side of a fluidic chamber with the nanostructures and subsequent layers facing down towards the solution, as seen in Fig. 3 (c). This placement reduces scattering of incident light at the cover glass and solution. The fluidic chamber is filled with a chloroauric acid (HAuCl_4_) solution. The solution is prepared by mixing hydrogen tetrachloroaurate(III) trihydrate (abcr GmbH, 16961-25-4, 99.99% metal basis, 49.5% Au) with deionized water (DI) in a ratio of 1 mg per 4 ml of water and is used as the precursor solution for gold deposition^40^.

The incident UV light excites electrons from the valence to the conduction band of the TiO_2_. These photogenerated electrons reduce metal precursors (Au^3+^ to Au^0^) from the solution, initiating nanoparticle nucleation on the surface of the TiO_2_. As growth continues plasmonic gold nanoparticles further enhance photocatalytic activity through near-field enhancement^41^ and additionally surface plasmon resonance (SPR) effects contribute to localized growth near nucleation areas^42,43^. Kinetic models, such as the Finke–Watzky two-step mechanism, describe gold nanoparticle formation via slow continuous nucleation followed by rapid autocatalytic surface growth, where existing particles catalyze further gold ion reduction on their surfaces^44,45^. Growing continuous gold lines via resonant excitation benefits greatly by this effect. While the non-structured TiO_2_ is capable of forming new nucleation sites on its surface, the gold particles from the solution are much more likely to cluster on existing ones. This is favorable when exciting resonantly as it further increases the contrast between high- and low-energy areas. The general chemical reaction mechanism can be described with^19^:

2 $$\:\mathrm{T}\mathrm{i}{\mathrm{O}}_{2}\left(e\right)+n\mathrm{A}{\mathrm{u}}^{3+}\underrightarrow{\varDelta\:}\mathrm{T}\mathrm{i}{\mathrm{O}}_{2}+n\mathrm{A}{\mathrm{u}}^{0}$$

3 $$\:{n\mathrm{A}\mathrm{u}}^{0}+{\mathrm{A}\mathrm{u}}^{3+}+\:\mathrm{T}\mathrm{i}{\mathrm{O}}_{2}\left(e\right)\to\:{\mathrm{A}\mathrm{u}}_{\mathrm{n}+1}^{0}+\mathrm{T}\mathrm{i}{\mathrm{O}}_{2}$$

Since there is no optical feedback for these samples and the nanostructure dimensions are small compared to the camera image, the alignment step of the laser is very important to ensure proper excitation of the area of interest. The fluidic chamber is then placed on the setup and excited by the laser operating at a power of 5 mW for 2 h. The incident excitation power on the sample was measured to be 1.822 mW. Afterward the excitation, the sample is rinsed with DI water and carefully dried with nitrogen. A schematic of the sample after the growth experiments is shown in Fig. 3 (b). Two grating periods are shown as an example that it results in different gold particle density on the nanostructures.

## Results and discussion

### Resonant photoluminescence enhancement

Fig. 4(a) and (b) show camera images of the sample’s surface at different positions of the nanostructure design, where Fig. 4 (a) shows the excitation of the fields C and Fig. 4 (b) shows a PL heatmap when exciting the hexagonal structures A. The original raw microscope image is provided in the Supplementary Fig. 4. The grating periods are given in Fig. 1 (a) and Fig. 1 (c). Both images show uniform blue emission from the DPVBi with localized resonant enhancements.

First, we observe the guided mode resonance (GMR) for the $\:{\Lambda\:}=220\:\mathrm{n}\mathrm{m}$ nanostructure under orthogonal white light excitation with a crossed polarizers configuration^46^. The GMR for this nanostructure is shown in red in Fig. 4 (c) and has a resonance wavelength of $\:{\lambda\:}_{\mathrm{r}\mathrm{e}\mathrm{s},220\mathrm{n}\mathrm{m}}\approx\:470\:\mathrm{n}\mathrm{m}$. We can now estimate the effective refractive index of the nanostructure with Eq. (1) and calculate the incident angle of the laser to match the excitation wavelength of the laser with the GMR of the nanostructured TiO_2_-DPVBi layer stack. The incident angle of the laser is set to $\:{\vartheta\:}_{\mathrm{i}\mathrm{n}\mathrm{c}}=30\:^\circ\:$. An increase in emission at specific nanostructures is observed from the images as the laser couples into the grating, increasing the field locally, leading to higher excitation inside the DPVBi layer. This local field enhancement is then monitored when exciting field C with the laser, as resonant coupling is observed by a strong PL increase of the nanostructured square with a grating period of $\:{\Lambda\:}=220\:\mathrm{n}\mathrm{m}$. The other eight nanostructured squares emit similar to the background as the resonance conditions aren’t fulfilled.

To provide a quantitative basis for the resonance condition associated with the strongest PL enhancement around $\:{\Lambda\:}=220\:\mathrm{n}\mathrm{m}$, we additionally estimated the expected resonant period using experimentally determined optical constants of our TiO_2_. Ellipsometry measurements on reference TiO_2_ films fabricated under identical conditions yield a refractive index of $\:{n}_{{\mathrm{T}\mathrm{i}\mathrm{O}}_{2}}\left(355\mathrm{n}\mathrm{m}\right)=3.156$ RIU (Supplementary Fig. 3). This refractive index is in good agreement with literature values^47^. Considering the effective guiding layer stack relevant for coupling at the nanostructured interface, we approximate an effective refractive index by a thickness-weighted average of the TiO_2_ (100 nm) and the photoresists layer (200 nm, $\:{n}_{\mathrm{A}\mathrm{m}\mathrm{o}\mathrm{n}\mathrm{i}\mathrm{l}}\left(355\mathrm{n}\mathrm{m}\right)\approx\:1.55$ RIU^48^ resulting in $\:{n}_{\mathrm{e}\mathrm{f}\mathrm{f}}\left(355\mathrm{n}\mathrm{m}\right)=2.085$ RIU. In this simplified estimate we neglect potential local indentation of the resist by the grating, assuming that the total resist volume is conserved. With these assumptions and Eq. (1), we obtain an expected resonant period of $\:{\Lambda\:}\approx\:224\:\mathrm{n}\mathrm{m}$. Although this estimate does not replace a full modal analysis of the corrugated stack, it provides a consistent order-of-magnitude confirmation of the resonance assignment, supporting our interpretation that the PL increase is driven by resonant coupling in the grating stack.

The hexagonal structures were excited similarly but the microscope image was processed to highlight local intensity enhancements. The relative background-normalized fluorescence intensity is shown as a heatmap in Fig. 4 (b). This analysis reveals clear resonant emission enhancements for grating periods $\:{\Lambda\:}>190\:\mathrm{n}\mathrm{m}$, with the strongest increase of approximately $\:10\%$ observed for $\:{\Lambda\:}=210\:\mathrm{n}\mathrm{m}$. This trend matches the peak in period-dependent emission observed in Fig. 4 (a), where $\:{\Lambda\:}=220\:\mathrm{n}\mathrm{m}$ yielded the highest integrated PL signal. This apparent discrepancy is explained by the substantially lower number of grating repetitions within the hexagonal lattice. Reducing the number of grating periods decreases the resonator quality factor and correspondingly broadens the resonance linewidth, thereby relaxing the resonance conditions enabling coupling across a wider spectral range^49,50^.

The processed fluorescence heatmap shows that the emission intensity closely follows the underlying TiO₂ nanostructure and appears only for grating periods within the expected resonance range^51^. This spatial correspondence indicates that the enhancement originates from localized excitation fields associated with the guided-mode resonance rather than from changes in emission directivity or lifetime^52^. Similar spatially patterned fluorescence enhancement has been reported for resonant waveguide gratings and photonic crystal surfaces and is widely used as a probe of resonant near-field distributions^28^. In line with this picture, our measurements confirm that the guided-mode resonance concentrates optical energy in the vicinity of the TiO₂ grating.

This experiment showed via an optical probe that local field enhancements at the nanostructured areas are possible when exciting with a monochromatic laser under the correct resonance conditions. We use these field enhancements to further observe localized gold growth on TiO_2_ without an additional emissive layer.

### Gold growth on nanopatterned substrates

**Fig. 5:**
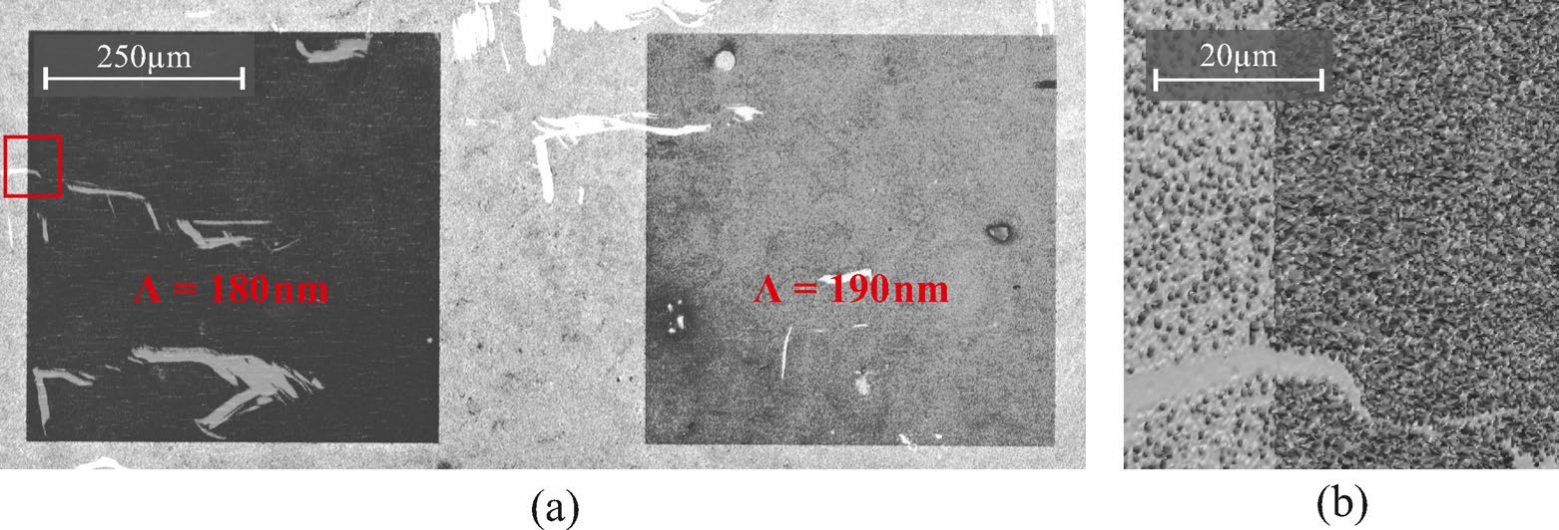
Laser interference microscope images of field C after 2 h of gold growth under laser excitation. **(a)** Magnification of two nanostructures with $\:{\Lambda\:}=180\:\mathrm{n}\mathrm{m}$ (left) and $\:{\Lambda\:}=190\:\mathrm{n}\mathrm{m}$ (right); the left nanostructure shows dense coverage with gold particles. **(b)** 3D-height scan of the 180-nm nanostructure at the boundary to planar TiO_2_. The position on the substrate is marked with the red square in **(a)**. Compared to the optical characterization, the grating period that resonantly couples incident light to the waveguide is lower with $\:{\Lambda\:}=180\:\mathrm{n}\mathrm{m}$. This behavior is to be expected since the effective refractive index of the TiO_2_ nanostructures is higher than the effective refractive index of the multilayer structure with DPVBi, given that their respective refractive index at $\:\lambda\:=355\:\mathrm{n}\mathrm{m}$ is $\:{n}_{{\mathrm{T}\mathrm{i}\mathrm{O}}_{2}}=3.156\:\mathrm{R}\mathrm{I}\mathrm{U}$ and $\:{n}_{\mathrm{D}\mathrm{P}\mathrm{V}\mathrm{B}\mathrm{i}}\approx\:1.61\:\mathrm{R}\mathrm{I}\mathrm{U}$.

First, we investigate the gold growth on field C with the nanostructured square areas. The coupling into the waveguide is higher compared to the hexagonal or triangular structures, as it was shown in the optical characterization with the emissive layer. We take laser interference microscope images of the nanostructures before and after the laser excitation. Figure 5 (a) shows two nanostructured squares with grating periods of $\:{\Lambda\:}=180\:\mathrm{n}\mathrm{m}$ (left) and $\:{\Lambda\:}=190\:\mathrm{n}\mathrm{m}$ (right). It is seen that the particle density is increased drastically for the 180-nm nanostructure compared to the 190-nm nanostructure and planar TiO_2_. The surface of the nanostructure is almost completely covered with gold particles, but only isolated particles are seen on the planar TiO_2_ and only a slightly increased particle density is seen for $\:{\Lambda\:}=190\:\mathrm{n}\mathrm{m}$. The surface of the nanostructured and non-structured areas are partially scratched during the cleaning and drying process as well as handling the samples. Additionally, Fig. 5 (b) shows a 3D-scan of the edge between the 180-nm area and planar TiO_2_. The difference in height due to the agglomeration of gold particles is clearly seen further showing the increased photocatalytic activity on the nanostructure. SEM images comparing the gold coverage on the nanostructured fields with $\:{\Lambda\:}=180\:\mathrm{n}\mathrm{m}$ and $\:{\Lambda\:}=370\:\mathrm{n}\mathrm{m}$ are presented in the Supplementary Information (Supplementary Fig. 17,18).

**Fig. 6:**
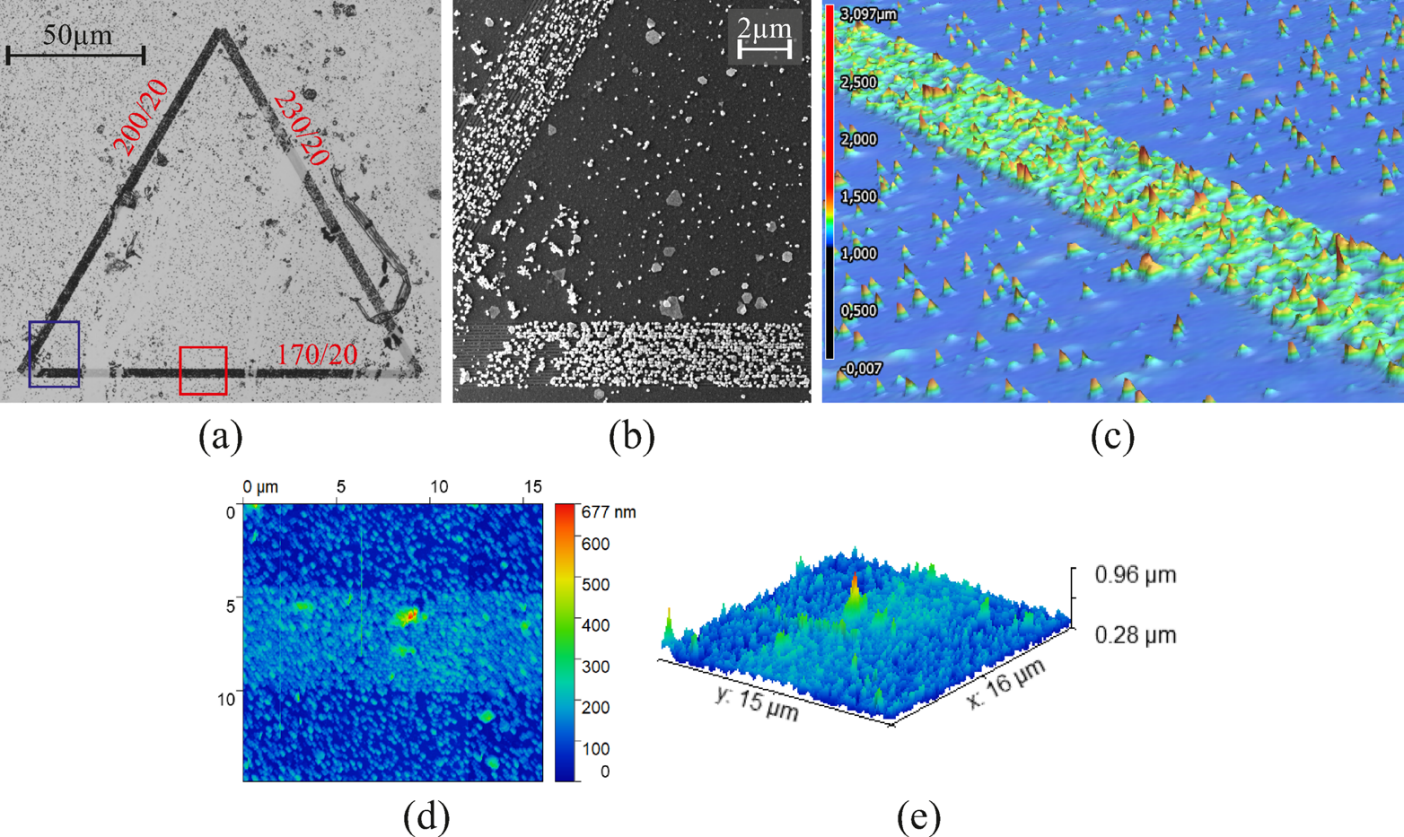
**(a)** Laser interference microscope image of field B with 50x magnification after 2 h of gold growth; grating periods and grating repetitions of the sides is denoted as $\:{\Lambda\:}/r$; sparse particle coverage on planar TiO_2_ while the nanostructures are covered seen as black lines. **(b)** SEM image of the corner between the 170-nm and 200-nm nanostructures; individual particles and the line formation on the nanostructures is seen. **(c)** 3D-height scan of the 170-nm nanostructure; the red box in **(a)** indicates the area used for the scan. **(d-e)** 2- and 3D AFM scans of the line under investigation.

Next, gold-growth experiments on the triangular structures on field B are carried out. The sides of the triangles each consist of a different grating period and the grating repetition is consistent within a triangle. We investigate the triangle with 170-nm, 200-nm and 230-nm gratings with a repetition of 20 each. Figure 6 (a) shows a laser interference microscope image of the triangle and a close-up SEM image of the corner between the 170-nm and the 200-nm grating is shown in Fig. 6 (b). The location is displayed by the blue rectangle in (a).

We observe that all three nanostructures of the triangle show increased photocatalytic activity of the TiO_2_ which led to a more dense formation of gold lines on the substrate. Getting resonant behavior for multiple grating periods is similar to what was shown earlier with the emission enhancements with the hexagonal structures. Due to the low quality factor of the resonance, the coupling condition is broadened, enabling resonant coupling and enhanced growth for several grating periods. Among these, the 170-nm grating exhibits the highest activity and the densest gold coverage, indicating that it is closest to the optimum resonance condition for our excitation wavelength and incident angle. For the 200-nm and 230-nm gratings, increased gold growth is observed but reduced significantly for larger grating periods. As an additional method of imaging the line formation, we record 3D-height profiles of the lines with the microscope. A cross section of the line with 170-nm grating is depicted in Fig. 6 (c). Single particles are scattered over the substrate and a dense continuous gold line is observed. This growth behavior is further studied and verified via AFM scans and SEM images of the gold lines. The continuous coverage is shown in Fig. 6 (b), where single particles are seen at non-structured areas but a layer of gold was grown above the nanostructures. 2D and 3D AFM scans are shown in Fig. 6 (d-e). It is notable that the resolution of the AFM scan is sufficient to show the 5 μm width of the line and even detects single particles.

This result is in good accordance to what was shown earlier. Within this broadened response, the 170-nm period shows the highest activity, which we attribute to the coupling of the laser into the waveguide. The energy increase due to this coupling inside the TiO_2_ enhances the photocatalytic growth properties, as more free charges can be generated that are available for gold particle formation on the surface. The increased surface of the nanostructures might influence the photocatalytic activity further, since the ridges are not present for planar areas. Created electrons might have a higher probability to diffuse to the surface of the TiO_2_ which again leads to a higher chance of nucleation. However, the experiment shows that the surface morphology on its own cannot be the decisive factor, as a similar particle density would be expected for other nanostructured areas.

**Fig. 7:**
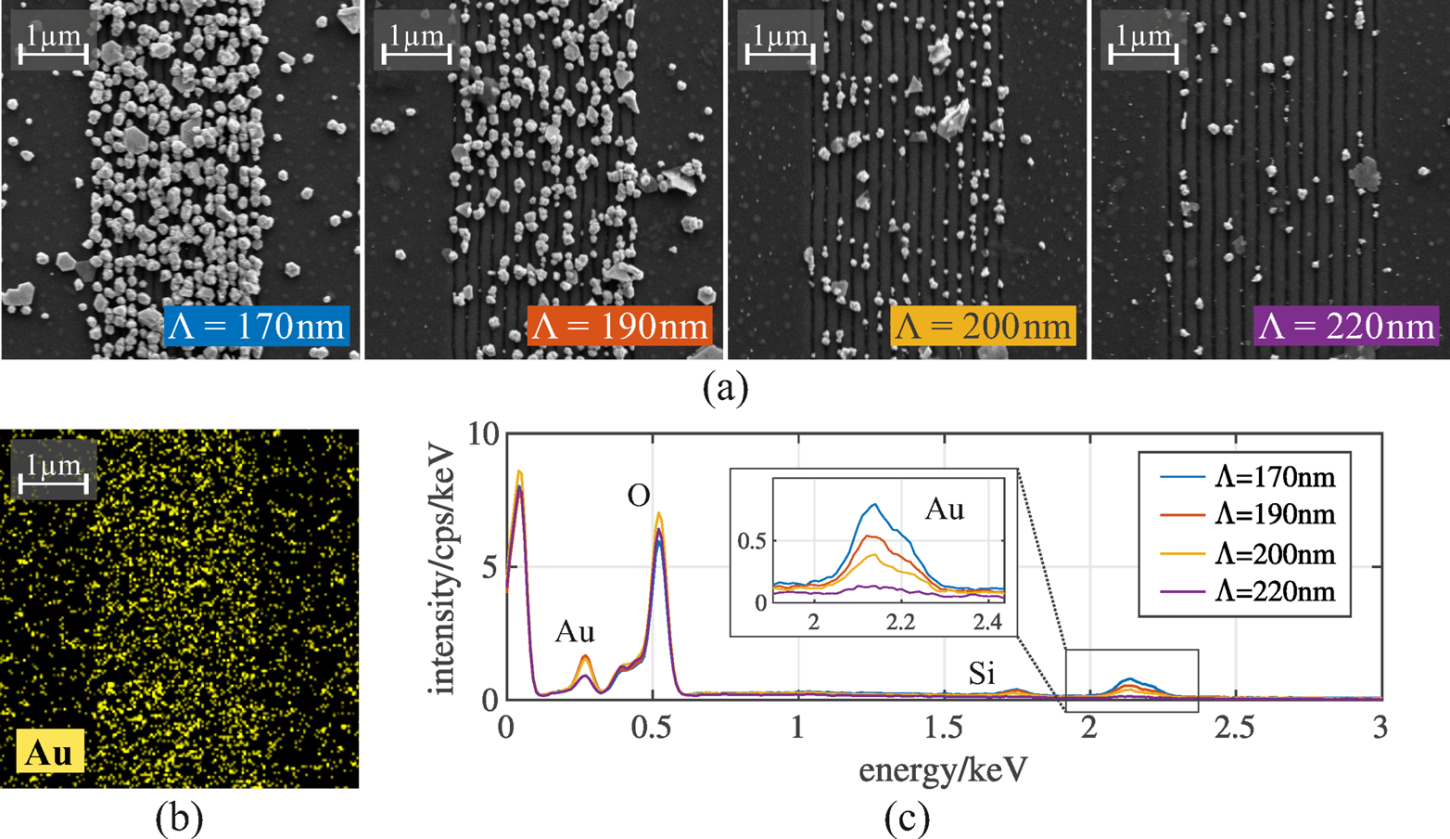
**(a)** SEM images of nanostructured lines with grating periods $\:{\Lambda\:}=170-220\:\mathrm{n}\mathrm{m}$ after 2 h of gold growth.

To investigate the grating-period dependent growth behavior more thoroughly, we repeated the growth experiment on the single nanostructured lines on field D. SEM images of the nanostructures after the gold growth are shown in Fig. 7 (a). Dense coverage of particles on the nanostructure is observed for the 170-nm grating. For larger grating periods of 190, 200, and 220 nm, the particle coverage gradually decreases until only very few particles remain visible. Consequently, the underlying nanostructure becomes increasingly apparent with each increase in grating period. We further performed an energy-dispersive x-ray (EDX) spectroscopy (Oxford Ultim Max; 15 kV) of the same positions to characterize the chemical composition of the particles that grew on the sample. An EDX map of gold (Au, yellow) is presented in Fig. 7 (b) and shows the gold coverage on the 170-nm grating. It can easily be seen, that in the middle of the map – the nanostructured TiO_2_ - the gold particles are dominant, whilst some gold particles are visible on the non-structured area left and right to the nanostructure, as expected from the leftmost SEM image in (a). The EDX spectra of the four SEM images with different grating periods are given in Fig. 7 (c). The inset shows a zoom at the gold Mα emission at roughly 2.12 keV. For shorter grating periods a signal is clearly visible, confirming the growth of gold particles on the surface. Further, it shows that with increasing grating period, the Au signal gets weaker, mirroring the particle coverage seen from the SEM images in (a). This trend is quantitatively supported by the wt% values extracted from the EDX maps, which decrease from 50.01 wt% (170 nm) to 39.97 wt% (190 nm), 29.69 wt% (200 nm), and 14.08 wt% (220 nm). The associated EDX maps for all grating periods, original SEM images and screenshots of the wt% values are included in the Supplementary Information (Supplementary Fig. 5–16).

This observation matches the theoretical kinetic behavior described in Sect. “Photocatalytic gold growth”. Without any nucleation sites the TiO_2_ is equally likely to form gold particles on its surface no matter if it is nanostructured or not. Increasing the field intensity through resonant coupling to the grating the nanostructured area then promotes nucleation which is followed by even more formation of gold particles next to it. This further proves that resonant excitation of the TiO_2_ leads to enhanced photocatalytic activity.

**Fig. 8:**
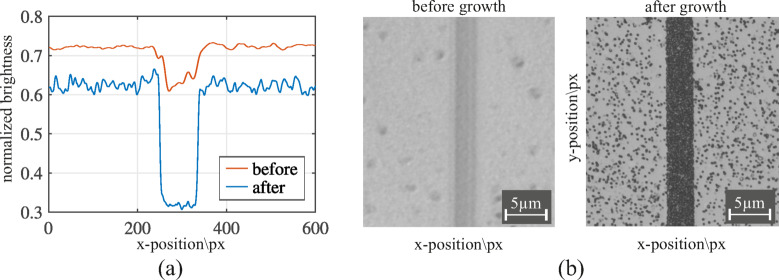
**(a)** Brightness normalized to the maximum background brightness of the microscope images **(b)** of the nanostructure before (red, left) and after (blue, right) the gold-growth experiment.

To quantify the gold density on the surface we process sections of the microscope images further. The region of interest is again the red square in Fig. 6 (b). We use the gray scale information of the images as a means to determine the density of the particles. These are displayed as black pixels in the microscope image which is recorded in reflection. Areas without growth are recorded in white/gray. The gray values of individual pixels of the images are summed up in y-direction, divided by the number of pixels, normalized to the maximum brightness and then plotted over the x-direction in pixels as shown in Fig. 8 (a). We compare the same area with images before the growth experiments, because we saw that the nanostructure itself is visible in the image as a darker hue of gray compared to planar TiO_2_. An image of the nanostructure before and after the growth experiment is depicted in Fig. 8 (b).

The evaluation of accumulated brightness over the nanostructure shows the expected result. Before gold particles are grown on the substrate the overall brightness is highest for the planar areas, reducing a little where the nanostructure is visible. The difference in intensity is approximately 10%. After the growth experiment the baseline intensity of planar areas is reduced by roughly 10% due to scattered particles on the surface. On the nanostructure the intensity is half compared to off the nanostructure indicating dense particle formation. Considering the percentile brightness decrease of the nanostructure itself, this shows that the majority of visible difference is due to the grown particles on the surface.

## Conclusions

In this work we presented an approach to influence photocatalytic gold growth by light-stimulus dependent excitation of nanostructured TiO_2_. Local field enhancements are achieved through guided-mode resonance effects in grating geometries. Using an angular excitation setup with a monochromatic laser, we systematically tuned resonant coupling into the nanostructures and correlated optical observations with post-growth characterization.

We designed and fabricated nanooptical templates to investigate the impact of grating parameters on resonant behavior and on the formation of metal-line networks. Resonant coupling was first verified using an optical probe layer. With 150 nm DPVBi on 100 nm TiO_2_ grating structures and excitation at $\:{\lambda\:}_{\mathrm{i}\mathrm{n}\mathrm{c}}=355\:\mathrm{n}\mathrm{m}$ and $\:{\vartheta\:}_{\mathrm{i}\mathrm{n}\mathrm{c}}=30^\circ\:$, resonant PL enhancement depends on the grating geometry: in the square structures, enhancement is most pronounced for $\:{\Lambda\:}=220\:\mathrm{n}\mathrm{m}$, whereas the hexagonal structures show enhancement over a broader period range ($\:{\Lambda\:}>190\:\mathrm{n}\mathrm{m}$). We attribute this broadening to the reduced number of grating repetitions in the hexagonal geometry.

Second, we performed resonant gold growth on the TiO_2_ templates without the emissive layer. We excited the TiO_2_ in a fluidic chamber with chloroauric acid solution for 2 h at an incident angle of $\:{\vartheta\:}_{\mathrm{i}\mathrm{n}\mathrm{c}}=30^\circ\:$. Laser interference microscopy, SEM and EDX reveal dense formation of gold lines, after resonant excitation, while non-resonant illumination leads to a more scattered distribution of gold particles on the TiO_2_ surface due to its inherent photocatalytic activity. Across both the optical probe experiments and the photoreduction results, a reduced number of grating repetitions lowers the resonance quality factor and broadens the coupling window, explaining why PL enhancement and growth is observed over a wider range of grating periods in low-repetition geometries. Within this broadened response, the strongest gold-line formation is observed for the 170-nm grating structures, consistent with the resonance conditions of the layer stack under our excitation geometry.

## Supplementary Information

Below is the link to the electronic supplementary material.

Supplementary Material 1

## Data Availability

Data underlying the results presented in this paper are not publicly available at this time and can be obtained from the corresponding author Jan Schardt upon reasonable request.
