# Supplementary material for: Resonant laser excitation for nanoscale photocatalytic gold growth on patterned templates
Source: Sci Rep. 2026 Jan 19;16:2592. doi: 10.1038/s41598-026-36556-5 (PMC12820206; doi:10.1038/s41598-026-36556-5)
Supplement: Supplementary file 1 — Supplementary Material 1 [file 41598_2026_36556_MOESM1_ESM.docx]

Supplementary Information

Resonant laser excitation for nanoscale photocatalytic gold growth on patterned templates

Jan Schardt^1,*^, Moritz Paulsen^1^, Fatemeh Abshari^1^, and Martina Gerken^1^

^1^ Integrated Systems and Photonics, Faculty of Engineering, Kiel University, Germany
* [jsch@tf.uni-kiel.de](mailto:jsch@tf.uni-kiel.de)

**Table of contents**

[Supplementary Fig. 1 AFM line scan across a 170-nm grating. 2](#_Toc216279385)

[Supplementary Fig. 2 FESEM scan of a 170-nm grating. 2](#_Toc216279386)

[Supplementary Fig. 3 Real (blue, left axis) and Imaginary part (red, right axis) of the refractive index of TiO_2_ measured via ellipsometry. 3](#_Toc216279387)

[Supplementary Fig. 4 Fluorescence Microscope image of the hexagonal nanostructures excited with a UV-Laser under 30°. 3](#_Toc216279388)

[Supplementary Fig. 5 SEM image of 170-nm grating after 2h of gold growth. 4](#_Toc216279389)

[Supplementary Fig. 6 EDX map of 170-nm grating. 4](#_Toc216279390)

[Supplementary Fig. 7 Weight Percentage Distribution of the EDX map of the 170-nm grating. 4](#_Toc216279391)

[Supplementary Fig. 8 SEM image of 190-nm grating after 2h of gold growth. 5](#_Toc216279392)

[Supplementary Fig. 9 EDX map of 190-nm grating. 5](#_Toc216279393)

[Supplementary Fig. 10 Weight Percentage Distribution of the EDX map of the 190-nm grating. 5](#_Toc216279394)

[Supplementary Fig. 11 SEM image of 200-nm grating after 2h of gold growth. 6](#_Toc216279395)

[Supplementary Fig. 12 EDX map of 200-nm grating. 6](#_Toc216279396)

[Supplementary Fig. 13 Weight Percentage Distribution of the EDX map of the 200-nm grating. 6](#_Toc216279397)

[Supplementary Fig. 14 SEM image of 220-nm grating after 2h of gold growth. 7](#_Toc216279398)

[Supplementary Fig. 15 EDX map of 220-nm grating. 7](#_Toc216279399)

[Supplementary Fig. 16 Weight Percentage Distribution of the EDX map of the 220-nm grating. 7](#_Toc216279400)

[Supplementary Fig. 17 SEM image of the edge between the 180-nm grating fields a planar TiO_2_ after 2h of gold growth. 8](#_Toc216279401)

[Supplementary Fig. 18 SEM image of the edge between the 370-nm grating fields a planar TiO_2_ after 2h of gold growth. 8](#_Toc216279402)

[Supplementary Fig. 19 Raw data (blue) of the XRD measurement. Smoothed data and the baseline, which is used for post-processing are shown in red and yellow, respectively. 9](#_Toc216279403)

Atomic force microscopy (AFM) measurements of the Λ = 170 nm grating were performed by Kelvin Nanotechnology Ltd using a [Model Name] system. The height profile extracted from a line scan across multiple periods reveals a grating depth of 38 nm, which is slightly larger than the nominal design value. These measurements confirm that the fabricated structures possess a well-defined periodic profile with uniform depth across the measured area.


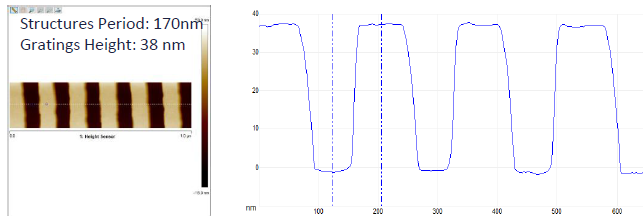


Supplementary Fig. 1 AFM line scan across a 170-nm grating.

Scanning electron microscope (SEM) image of the Λ = 170 nm recorded using a [Model Name] by Kelvin Nanotechnology Ltd. shows the morphological quality of the nanostructures with a fill factor of $\approx45\%$.


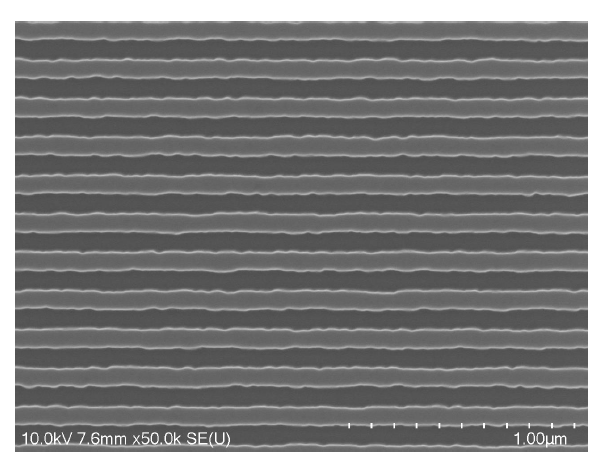


Supplementary Fig. 2 FESEM scan of a 170-nm grating.

Refractive index measurements of TiO_2_ via ellipsometry. The refractive index for the given TiO2 layers at $\lambda=355 \mathrm{nm}$ is 3,156 RIU.


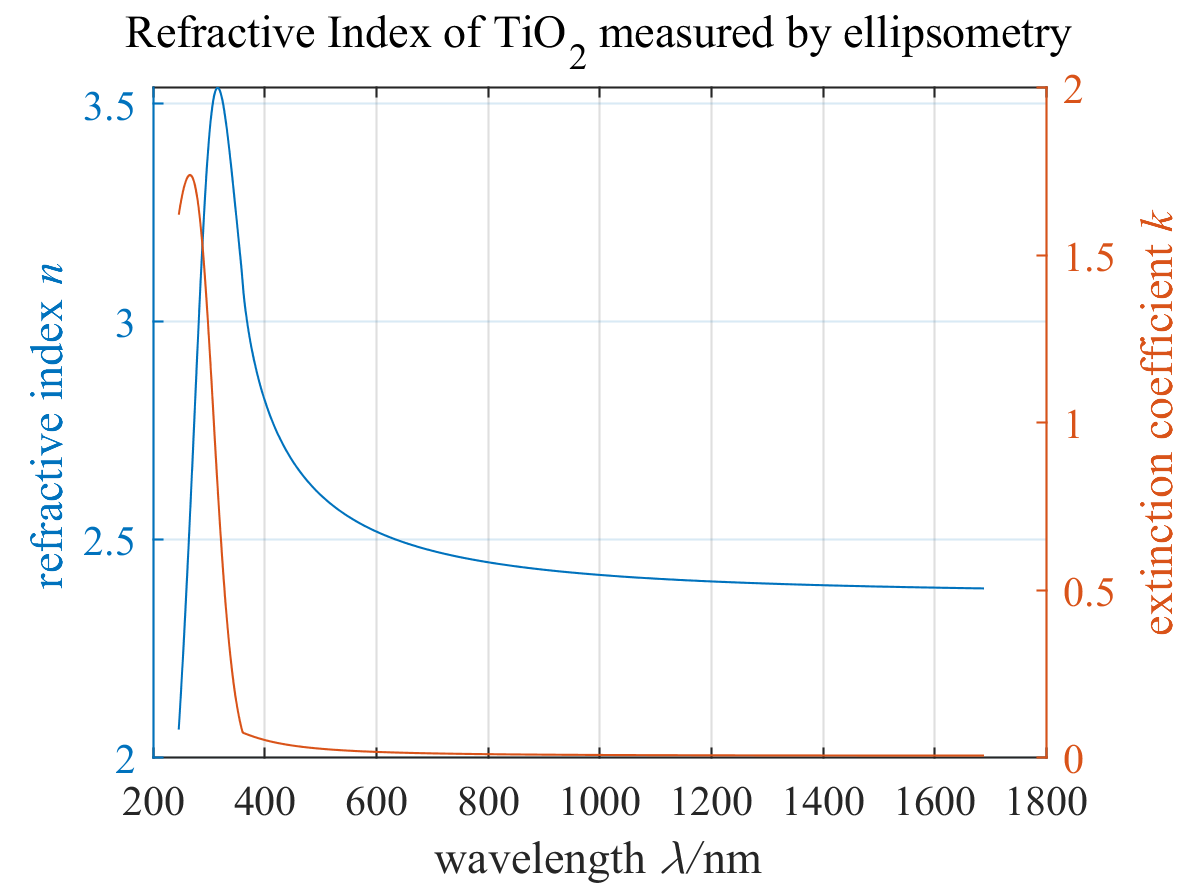


Supplementary Fig. 3 Real (blue, left axis) and Imaginary part (red, right axis) of the refractive index of TiO_2_ measured via ellipsometry.

Original, non-processed microscope image of the fluorescent DPVBi layer with hexagonal nanostructures beneath, excited with a UV-laser under 30°. The processed image is shown in Fig. 4 (b).


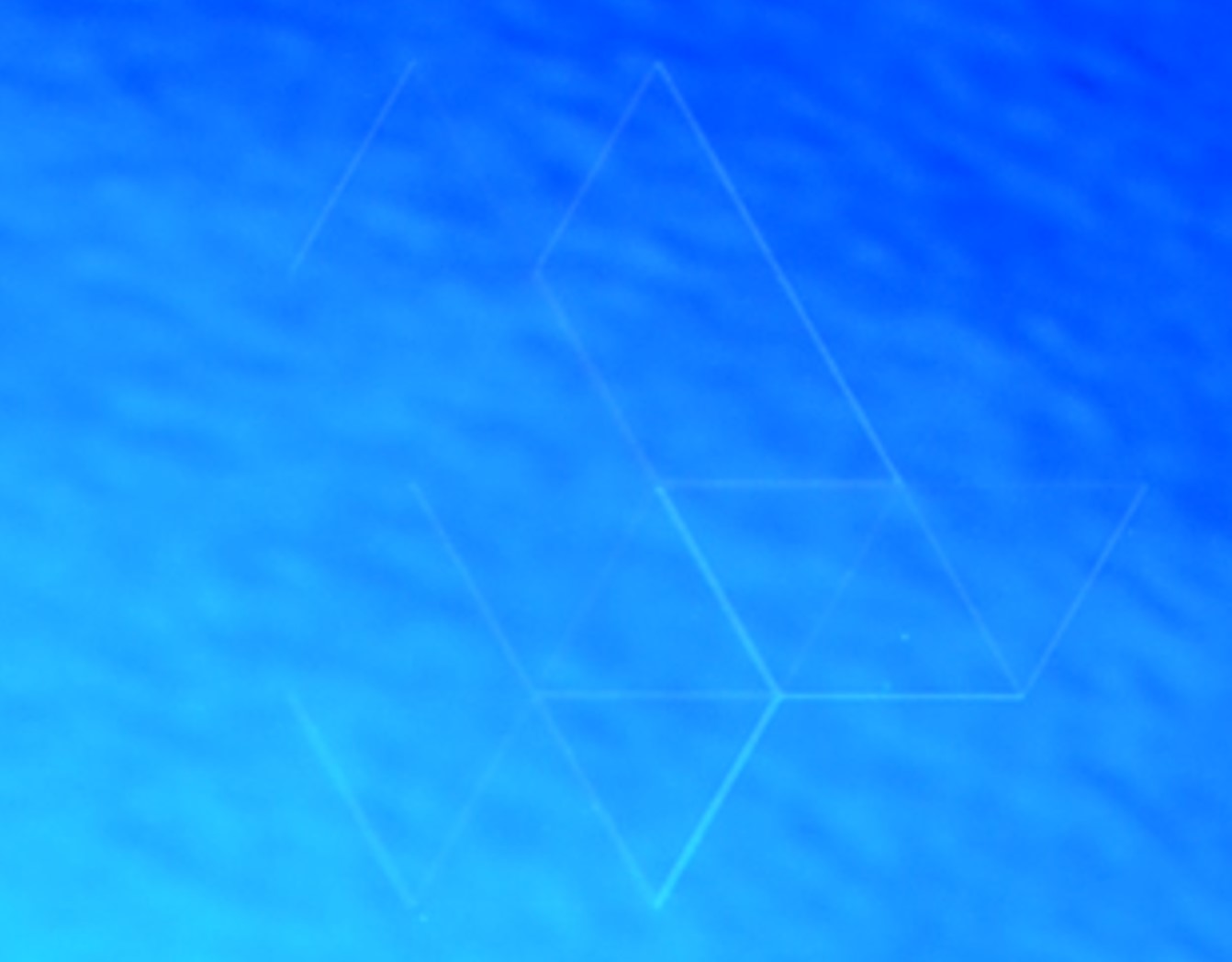


Supplementary Fig. 4 Fluorescence Microscope image of the hexagonal nanostructures excited with a UV-Laser under 30°.

SEM images and EDX maps of nanostructured lines (field D) after 2h of gold growth.

$\Lambda=170 \mathrm{nm}$:





Supplementary Fig. 5 SEM image of 170-nm grating after 2h of gold growth.


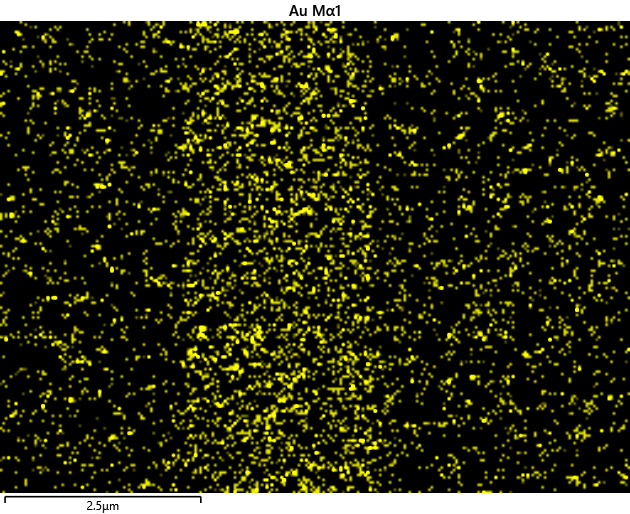


Supplementary Fig. 6 EDX map of 170-nm grating.

Wt%:


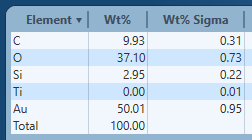


Supplementary Fig. 7 Weight Percentage Distribution of the EDX map of the 170-nm grating.

$\Lambda=190 \mathrm{nm}$:





Supplementary Fig. 8 SEM image of 190-nm grating after 2h of gold growth.


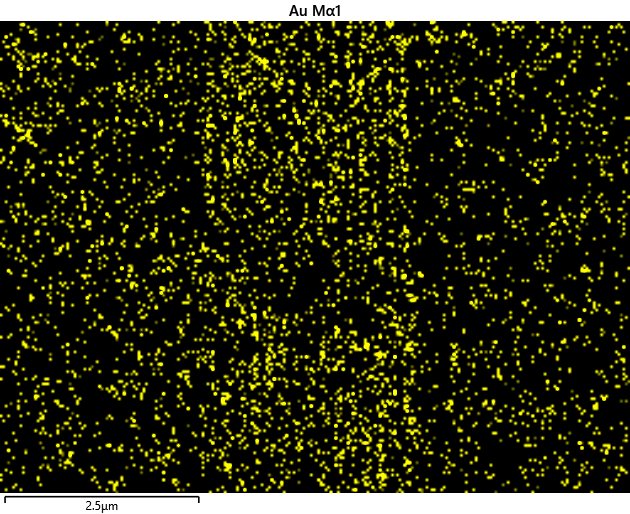


Supplementary Fig. 9 EDX map of 190-nm grating.

Wt%:


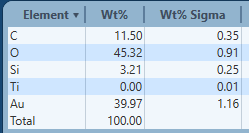


Supplementary Fig. 10 Weight Percentage Distribution of the EDX map of the 190-nm grating.

$\Lambda=200 \mathrm{nm}$:





Supplementary Fig. 11 SEM image of 200-nm grating after 2h of gold growth.


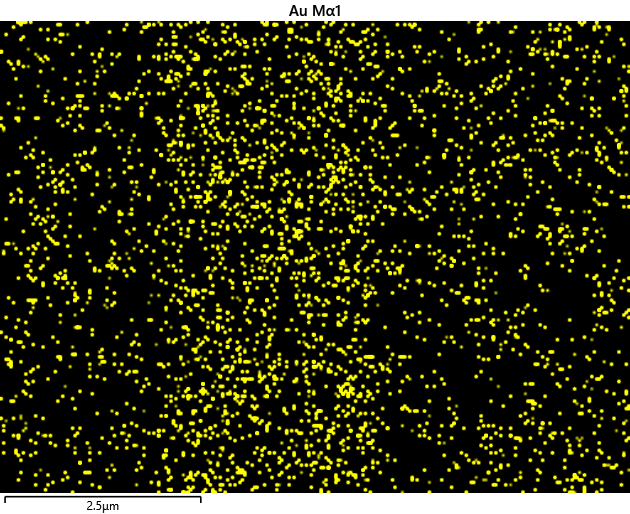


Supplementary Fig. 12 EDX map of 200-nm grating.

Wt%:


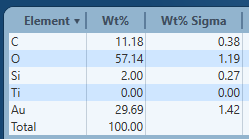


Supplementary Fig. 13 Weight Percentage Distribution of the EDX map of the 200-nm grating.

$\Lambda=220 \mathrm{nm}$:





Supplementary Fig. 14 SEM image of 220-nm grating after 2h of gold growth.


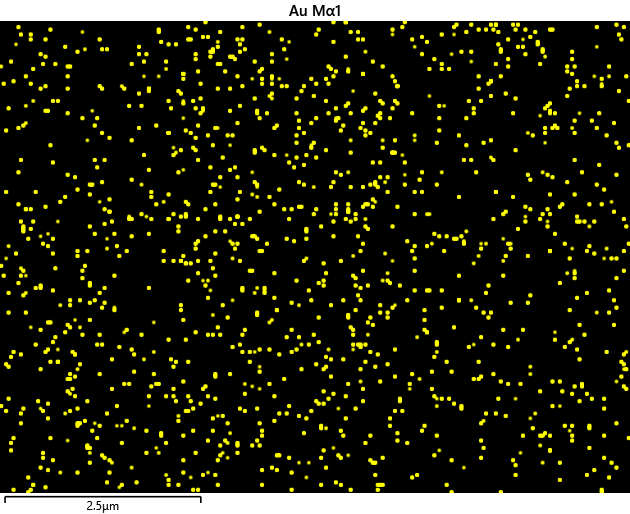


Supplementary Fig. 15 EDX map of 220-nm grating.

Wt%:


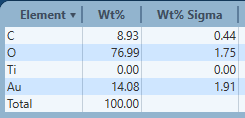


Supplementary Fig. 16 Weight Percentage Distribution of the EDX map of the 220-nm grating.

SEM images of nanostructured squares (field C) after gold growth:

Growth on nanostructure with $\Lambda=180 \mathrm{nm}$:





Supplementary Fig. 17 SEM image of the edge between the 180-nm grating fields a planar TiO_2_ after 2h of gold growth.

Growth on nanostructure with $\Lambda=370 \mathrm{nm}$:





Supplementary Fig. 18 SEM image of the edge between the 370-nm grating fields a planar TiO_2_ after 2h of gold growth.

Raw date, smoothed data and a baseline correction fit for the XRD measurement of TiO_2_ layer after annealing at 400°C for 90 minutes.


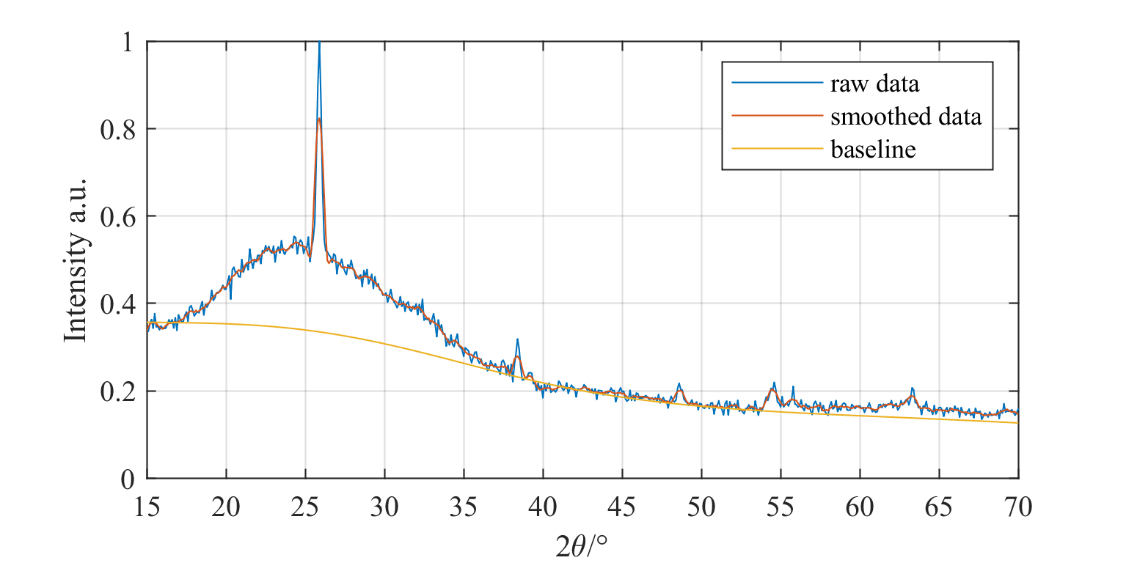


Supplementary Fig. 19 Raw data (blue) of the XRD measurement. Smoothed data and the baseline, which is used for post-processing are shown in red and yellow, respectively.
